# Role of miRNA-19a in Cancer Diagnosis and Poor Prognosis

**DOI:** 10.3390/ijms22094697

**Published:** 2021-04-29

**Authors:** Alessio Ardizzone, Giovanna Calabrese, Michela Campolo, Alessia Filippone, Dario Giuffrida, Francesca Esposito, Cristina Colarossi, Salvatore Cuzzocrea, Emanuela Esposito, Irene Paterniti

**Affiliations:** 1 Department of Chemical, Biological, Pharmaceutical and Environmental Sciences, University of Messina, Viale Ferdinando Stagno D’Alcontres, 98166 Messina, Italy; aleardizzone@unime.it (A.A.); gcalabrese@unime.it (G.C.); campolom@unime.it (M.C.); alessia.filippone@unime.it (A.F.); salvator@unime.it (S.C.); ipaterniti@unime.it (I.P.); 2 Istituto Oncologico del Mediterraneo, Via Penninazzo 7, 95029 Viagrande, Italy; dario.giuffrida@grupposamed.com (D.G.); cristina.colarossi@grupposamed.com (C.C.); 3 IOM Ricerca Srl, Via Penninazzo 11, 95029 Viagrande, Italy; francesca.esposito@grupposamed.com

**Keywords:** cancer, miRNAs, miR-19a, tumor suppressors, oncogene, diagnostic and prognostic markers, poor prognosis, miR-19a therapeutic value

## Abstract

Cancer is a multifactorial disease that affects millions of people every year and is one of the most common causes of death in the world. The high mortality rate is very often linked to late diagnosis; in fact, nowadays there are a lack of efficient and specific markers for the early diagnosis and prognosis of cancer. In recent years, the discovery of new diagnostic markers, including microRNAs (miRNAs), has been an important turning point for cancer research. miRNAs are small, endogenous, non-coding RNAs that regulate gene expression. Compelling evidence has showed that many miRNAs are aberrantly expressed in human carcinomas and can act with either tumor-promoting or tumor-suppressing functions. miR-19a is one of the most investigated miRNAs, whose dysregulated expression is involved in different types of tumors and has been potentially associated with the prognosis of cancer patients. The aim of this review is to investigate the role of miR-19a in cancer, highlighting its involvement in cell proliferation, cell growth, cell death, tissue invasion and migration, as well as in angiogenesis. On these bases, miR-19a could prove to be truly useful as a potential diagnostic, prognostic, and therapeutic marker.

## 1. Introduction

Cancer is one of the most common diseases affecting millions of people worldwide every year, representing the second leading cause of mortality after cardiovascular disease [1]. Uncontrolled cell growth due to genetic alterations [2], environmental factors [3] such as smoking [4], incorrect diet [5], obesity [6], infections [7], ionizing radiation [8], stress [9] and environmental pollutants [10] are the common denominators of each type of neoplasm. Especially, genetic alterations, which include chromosomal abnormalities and genetic mutations, represent one of the most noticeable causes [11] that contribute to tumorigenesis, affecting cell growth and metastases development [12].

Currently, although many conventional and innovative therapies are a valuable aid in the fight against cancer, the mortality rate remains high; in this context, late diagnosis represents one of the unfavorable factors [13]. Therefore, it is assumed that a precise and accurate search for diagnostic biomarkers can represent a turning point for cancer treatment by decreasing the poor prognosis in patients [14].

In the last decade, the diagnostic, prognostic, and therapeutic properties of microRNAs (miRNAs) have been investigated in many cancer types [15]. In fact, much evidence has demonstrated that miRNAs play important roles in tumorigenesis, development, and clinical therapy by acting as oncogenes or tumor-suppressor genes in various cellular processes such as tumor proliferation, apoptosis, angiogenesis, invasion, and metastasis [16].

Molecularly, miRNAs are small, endogenous, single-stranded, non-coding RNA molecules, approximately 20–22 nucleotides in length that originate from a stem-loop precursor [17].

The synthesis of mature miRNAs is a process that begins in the cell nucleus and completes in the cytoplasm, also involving highly specific enzymes [18]. Most miRNAs are transcribed by the RNA polymerase II complex, or to a lesser extent by RNA polymerase III [19], in a long primary polyadenylated transcript called pri-miRNA (100–1000 nt). The monocistronic pri-miRNA comprises a 7-methylguanosine cap at the 5′ end and a poly (A) tail at the 3′ end folded into a hairpin secondary structure consisting of a double-stranded stem, a erminal loop and two single-stranded segments parallel to the 3′ and 5′ ends [18]. The pri-miRNA, inside the cell nucleus, is split into smaller molecules (about 70–80 nucleotides), called pre-miRNA, from a very specific complex composed of the enzymes DGCR8 and Drosha (type III RNA-ase). The cut made by DGCR8 must be very precise to ensure the affinity of the miRNA for the target mRNA [18]. Successively, the pre-miRNA is transported into the cytosol by Exportin-5 protein (nucleus–cytoplasmic transporter) and Ran–Guanosine Triphosphate (Ran-GTP) cofactor and further cleaved by Dicer (type III RNase) [18] in a mature, short, double-stranded RNA (dsRNA) of about 22 nt. The dsRNA is bound by the Argonauta protein (Ago) and incorporated into the enzymatic complex called RISC (RNA-induced silencing complex), as a mature miRNA exercising its biological function [18].

miRNAs induce gene silencing by overlapping complementary sequences present on mRNA molecules; this overlap involves the repression of messenger translation and its degradation [20]. The silencing operated by an miRNA can occur through several mechanisms which may include the cleavage of the RNA molecule, or the destabilization of the RNA molecule by reducing the length of the polyA tail, or the decrease in the translation efficiency of the RNA molecule [21].

Most human miRNAs reside in the introns of genes or noncoding mRNA transcript regions [22], while other miRNAs are found within 3′ UTRs of mRNA genes, exons of noncoding mRNA genes, or are clustered with other miRNA genes [23]. miRNAs are evolutionarily conserved from worms to humans, and on the basis of the sequence homology at the 5′ end of the mature miRNAs can be clustered into families. A single miRNA can regulate as many as 200 gene targets different in their function, such as transcription factors, secreted factors, receptors, and transporters [24].

Thus, miRNAs form a complex monitoring network which, by negatively regulating the expression of their target genes at the post-transcriptional level, can control a wide variety of physiological functions [25].

Precisely for these properties, miRNAs have aroused considerable interest as prognostic markers and therapeutic targets for human neoplasms [26].

The members of the miR-19 family have identical seed regions and arise from two different paralogous clusters, miR-17-92 (miR-19a and miR-19b-1) and miR-106a-363 (miR-19-b-2) [27].

The miR-19 family plays an important role in regulating and maintaining tissue homeostasis and the normal development of organisms [27,28]. It has also been found that the miR-19 family contributes to the homeostatic maintenance of the immune system, specifically lymphocytes, the regulation of the differentiation of follicular helper T cells [29], and the modulation of the development of B cells [30]. Some evidence suggests that the miR-19 family is implicated in regulating inflammation, tissue fibrosis, aging, metabolism, and tumorigenesis [27].

Moreover, members of the miR-19 family contribute to regulating the development of the nervous system, respiratory systems, cardiovascular systems, blood vessel formation, vertebrate axis, etc. Accordingly, their dysregulation often results in various diseases, and even cancer [27].

Among miR-19 family members, miR-19a is the most well-known oncogenic miRNA [31], and its oncogenic activity results in promoting c-MYC-induced lymphomagenesis by repressing apoptosis and the tumor suppressor phosphatase and tensin homolog (PTEN) [31]. Furthermore, miR-19a activates the protein kinase B (AKT)-mammalian target of rapamycin (mTOR) pathway, thereby functionally antagonizing PTEN to promote cell survival [31].

PTEN targeted by miR-19a [32], and more specifically of PTEN/AKT signaling [33,34], also induces tumor cells’ resistance to chemotherapeutic agents. Chemoresistance is the primary cause of treatment failure in cancer patients [35] and it is attributed to various aspects, including diminished drug accumulation and drug–target interaction, increased tumor stem cell populations and autophagic activity, and reduced apoptotic processes in cancer cells [35].

miR-19a has been shown to alter cellular sensitivity to chemotherapy drugs, including the well-known cisplatin, 5-Fluorouracil and Adriamycin, and to upregulate the expression of P-glycoprotein (P-gp), critical in the management of cytotoxic drug efflux [36].

Therefore, it is also clear that in this circumstance, the control of miR-19a levels could contribute to positive outcomes for cancer patients.

Moreover, several studies have identified thousands of circular RNAs (circRNAs) in various organisms, denoting their importance in various pathophysiological processes [37]. Indeed, circRNAs have the ability to regulate gene expression by affecting transcription, mRNA turnover, and also translation by sponging RNA-binding proteins and miRNAs [37].

Consequently, given the wide correlation between circRNAs on miRNA activity, several studies, especially in the oncology field, have investigated the impact of miRNA sponging by circRNA on gene regulation [38,39]. In this context, innovative methods for its molecular detection can play a key role in multiple identifications and data correlation, providing important oncology research advancements.

Hence, it is established that miR-19a is involved in some processes such as carcinogenesis, tumor progression, and chemoresistance by the modulation of several signaling pathways. Therefore, based on all the evidence previously presented, this review aims to summarize the current status and knowledge of the predictive role of miR-19a in different types of cancer and its potential clinical relevance for cancer diagnosis and prognosis.

## 2. Roles and Mechanisms of Action of miR-19a in Clinical Cancer Features

Recently, scientific evidence has indicated the dysregulation of miRNAs in cancer initiation, progression, and aggressiveness, thus affecting the clinical features of cancer patients.

On this basis, we focused on miR-19a, probing its molecular mechanisms and analyzing its key role in different tumor types.

Several studies have shown that aberrantly expressed miRNAs contribute to the initiation and progression of brain tumors; between these, miR-19a has a central role.

Qin et al. [40] showed that the upregulation of miR-19a-3p promoted cell proliferation, migration, and invasion by repressing the expression of PTEN, as reported in Table 1. PTEN is one of the most frequently mutated tumor suppressor genes [41] in human cancers that plays a key role in tumor cell growth, survival, and metabolic regulation. Functionally, PTEN acts as a negative regulator of cell survival and protein synthesis via inhibition of the phosphatidylinositol 3-kinase (PI3K)/AKT [41], one of the most important molecular pathways involved both in cell survival and in malignant neoplasms, which contributes, if altered or deregulated, to tumor pathogenesis [42] and chemoresistance [43].

On other hand, Chen et al. [44] also reported that miR-19a-3p overexpression promotes cell proliferation and invasion by targeting RhoB in glioma, while its inhibition suppresses them, suggesting that miR-19a may act as an oncogene in gliomas.

The link between the Rho family and miR-19a-3p is also elucidated by Lv et al. [45]. In their study, the authors identified the existence of a circ-EPB41L5/miR-19a/EPB41L5/RhoC/AKT regulatory axis. Circ-EPB41L5 inhibits the proliferation, migration, and invasion of glioma cells by sponging miR-19a-3p and regulating the host gene *EPB41L5* expression, which reduces the progression of glioma by inhibiting RhoC and p-AKT.

*RhoC* upregulation is associated with cell proliferation, contributing to the epithelial–mesenchymal transition (EMT). In addition, *RhoC* improves cell motility, which consequently results in a greater ability of the tumor to become invasive [46]. It is known that there is a connection between the increase in *RhoC* expression and an advanced stage of the tumor as well as with the presence of metastases [47]; to which certain crosstalk with angiogenic factors such as vascular endothelial growth factor (VEGF) also contributes [48].

Downregulation of miR-19a in gliomas plays an anti-oncogenic role, which suggests its potential application as a target for gene therapy. Furthermore, because its overexpression is often associated with a poor prognosis, it could represent a new diagnostic and prognostic marker for gliomas.

Xu et al. [49] investigated the impact of miR-19a-3p/miR-19b-3p on clinicopathologic factors and the prognosis of patients with ESCC. They observed that miR-19b-3p expression was positively correlated with tumor size, lymph node metastasis, and clinical stage, while miR-19a-3p is a prognostic indicator for progression-free survival and overall survival.

Similar results were obtained from Plum et al. [50]. They demonstrated that upregulation of miR-19a/b is associated with tumor progression and the occurrence of lymph node metastasis in human esophageal adenocarcinoma, indicating that miR-19a/b could represent a new prognostic biomarker in this cancer form.

Many studies [51,52,53] have shown that miR-19a is involved in the proliferation of human gastric cancer (GC). Yuan et al. [51] demonstrated that miR-17-92a-1 Cluster Host Gene (MIR17HG)-derived miR-18a and miR-19a-3p coordinately mediate GC cell metastasis by directly inhibiting mothers against decapentaplegic homolog 2 (SMAD2) expression and upregulating Wingless-related integration site (Wnt)/β-catenin signaling.

Qin et al. demonstrated that the suppressor of cytokine signaling 1 (*SOCS1*) is a novel target of miR-19a-3p in GC cells. They reported that miR-19a-3p expression is inversely correlated with *SOCS1* expression in GC cells, and that its overexpression markedly promotes proliferation and tumorigenicity both in vitro and in vivo [54]. In accordance with this evidence, other studies [55,56] have confirmed miR-19a overexpression in GC patients, suggesting that miR-19a could represent a potential new diagnostic biomarker for GC.

Liu et al. [57] demonstrated that miR-19a-3p is involved in colorectal cancer, promoting its proliferation and migration by targeting T cell Intracellular Antigen 1 (TIA1), thus also suggesting miR-19a as a new diagnostic and prognostic biomarker for gastrointestinal cancers.

The role of miR-19a-5p in hepatocellular carcinoma (HCC) has also been elucidated by Baik et al. [58], who showed that the suppression of adenine nucleotide translocase 2 (ANT2) by short hairpin RNA (shRNA) downregulates miR-19a through the PI3K/Akt pathway. The knockdown of ANT2 directly downregulates miR-19a, thus resulting in the suppression of tumor growth in HCC cells and clinical samples.

Tan et al. [59] demonstrated that high levels of miR-19a-5p correlate with poor prognosis in patients, proposing that miR-19a is a potential therapeutic target for pancreatic cancer.

Recently, it has been revealed that PLGF influences miR-19a-3p expression by modulating c-*MYC* [60]. Furthermore, a positive pairwise correlation among PLGF, c-*MYC*, and miR-19a expression in gallbladder cancer (GBC) tissues has been displayed [60]; this finding confirmed that the PLGF/c-MYC/miR-19a axis is involved in tumor progression of the gastrointestinal tract.

The upregulated expression of miR-19a-3p has also been determined in clinical tongue squamous cell carcinoma cells (TSCC) specimens [61].

Wu et al. [62] reported that miR-19a-3p is upregulated in laryngeal squamous cell carcinoma (LSCC) patients and is correlated with neck nodal metastasis, poor differentiation, and advanced stage, indicating that its overexpression is associated with reduced overall survival. It has also been demonstrated that miR-19a plays an influent effect in laryngeal verrucous squamous cell carcinoma (LVSCC), a highly differentiated form of LSCC. Marioni et al. [63] reported that miR-19a expression is significantly higher in malignant glottic lesions (LSCC and LVSCC) than in benign ones. Thus, these studies indicate the oncogenic role of miR-19a in the progression of LSCC and denote it as a possible biomarker to establish an earlier diagnosis as well as a marker of differentiation in the various forms of laryngeal tumors.

miR-19a overexpression is involved in the pathophysiology of lung cancer, and is associated with the poor prognosis, metastasis, and proliferation of pulmonary cancer cells [64]; thus, it constitutes a good biomarker and a possible target therapy for lung tumors [65].

Gu et al. [66] demonstrated that miR-19a-3p/miR-19b-3p promotes the proliferation and migration of lung cancer cells by targeting Microtubule-Associated Scaffold Protein 1 (MTUS1). Following these suggestions, miR-19a could represent an important diagnostic and prognostic marker for differential diagnoses of lung cancers [67].

Some clinical studies [68,69] have shown that high levels of miR-19a are implicated in a more frequently large tumor size, advanced clinical stage, positive distant metastasis, and poor response to chemotherapy in osteosarcoma patients. Huang et al. [70] and Zou et al. [71] described that miR-19a-5p and miR-19a-3p overexpression contributes to both the risk of poor prognosis in osteosarcoma and the probability of developing metastases, respectively.

miRNAs profiling can also be useful for the characterization and classification of different thyroid carcinomas, as well as in strengthening therapeutic strategies.

Calabrese et al. [72] showed that miR-19a-3p overexpression is correlated with a poor prognosis of thyroid cancers, highlighting its contribution to more de-differentiation and aggressiveness.

These results suggest that not only does miR-19a-3p have an important role in the malignancy of thyroid cancers [73], but it also represents an important prognostic indicator and is a good therapeutic target [74] for anaplastic thyroid carcinoma (ATC) patients.

The role of miR-19a has also been investigated in clear cell renal cell carcinoma (ccRCC) tissues and human cell lines, highlighting that the high expression of miR-19a-3p is correlated with poor prognosis via promoting cell proliferation and suppressing PTEN/mothers against decapentaplegic homolog 4 (SMAD4) expression [75]. In addition, Niu et al. [76] indicated that miR-19a-3p directly targets the 3′untranslated region (3′UTR) of RhoB, promoting tumorigenesis, cancer cell proliferation, and invasiveness, and suggesting the clinical potential of miR-19a as a molecular target in ccRCC. Ge et al. [77] assessed the relationship of high miR-19a levels with the progression and prognosis of chromophobe renal cell carcinoma (chRCC), demonstrating that miR-19a inhibition is significantly associated with both recurrence-free survival and overall survival.

miR-19a upregulation has also been correlated with bladder cancer prognosis. The role of the PI3K/AKT pathway and its crosstalk with miR-19a-3p in bladder carcinogenesis has also been elucidated by Calderaro et al. [78], thus opening the way to involvements between miR-19a and various molecular patterns in bladder urothelial carcinomas (UCs).

Mearini et al. [79] reported that miR-19a-3p is overexpressed in bladder cancer carcinogenesis and its oncogenic role is dependent on targeting PTEN [80] as well as inhibiting the expression of RhoB, in order to promote the invasion and EMT of bladder cancer cells [81]. Furthermore, it has been shown that miR-19a overexpression is associated with the poor prognosis of bladder cancer patients [82], representing a good starting point for future preclinical and clinical exploration.

Despite the initial controversy over the beneficial role of miR-19a in prostate cancer [83], recent studies are consistent in indicating its crucial role in prostate tumorigenesis and progression [84]. Its involvement includes the promotion of cell migration, invasion, and EMT in prostate cancer by directly binding to Cullin-5 (CUL5) mRNA 3′-UTR as reported by Wang et al. [85]; the regulation of proliferation and apoptosis of prostate cancer cells by targeting the B cell translocation gene 1 (BTG1) as described by Lu et al. [86], and the inhibition of vacuolar protein sorting-associated protein 37A (VPS37A) expression, as indicated by Fu et al. [87].

All these data highlight the importance of miR-19a for the development of new targeted therapies and suggest its use as a prognostic biomarker in prostate cancer patients [88].

miR-19a overexpression promotes cell proliferation, metastasis, migration, invasion, and angiogenesis in breast cancer (BC) [89]. Sochor et al. [90] developed and validated a composite risk score based on the expression of three miRNAs, including miR-19a, with prognostic value for BC. They reported that miR-19a overexpression is correlated specifically with bone metastasis, suggesting that it could be a good diagnostic and prognostic marker for BC. Additionally, Kawaguchi et al. [91] showed that miR-19a overexpression is correlated with poor prognosis and the risk of multiple metastases, as well as angiogenesis and EMT.

Alunni-Fabbroni et al. [92] indicated that miR-19a-3p shows a promising role differentiating early BC patients at different time points and from healthy controls. In addition, the regulation of miR-19a in BC is also useful in managing chemoresistance, as suggested by Liang et al. [32]. Ouchida et al. [93] suggested that inosine monophosphate dehydrogenase 1 (IMPDH1) and probable aminopeptidase-like 1 (NPEPL1) genes are direct targets of miR-19a in BC, while the exogenous expression of these genes is not associated with the growth suppression of MCF-7 cells. Hence, once again, the importance and usefulness of miR-19a both as a diagnostic biomarker and as a molecular target are highlighted.

Scientific findings have confirmed the involvement of miR-19a in malignant lymphoma, elucidating its crucial roles in the tumorigenesis and pathogenesis of aggressive transformed, high-grade, and refractory lymphomas, highlighting its prognostic role [94].

Lv et al. [95] performed bioinformatic analyses and demonstrated that 41 target genes of miR-19a are associated with the development and progression of multiple myeloma (MM), suggesting its potential role as a biomarker. Another study [96] also confirmed that miR-19a-3p plays the role of an oncogene by regulating the PTEN/AKT/pAKT pathway in MM and promoting cell proliferation and inhibiting apoptosis.

Wang et al. [97] demonstrated that miR-19a-3p is highly expressed in ovarian cancer tissues and cell lines and that its overexpression promotes proliferation, while its down-regulation reduces the growth of ovarian cancer cells. Furthermore, in this study, the authors suggested that the overexpression of PTEN suppresses miR-19a, promoting an effect on cancer cell growth, indicating that miR-19a expression and PTEN are inversely related in ovarian cancer tissues. The role of miR-19a has also been investigated in metastatic serous ovarian cancer (SOC). In fact, Wahab et al. [98] described a significant differential expression of 48 miRNAs, including miR-19a, in metastatic SOC compared to healthy subjects. These studies proved a potential oncogenic role of miR-19a in ovarian cancer, suggesting that it could represent a promising marker for ovarian cancer diagnosis, prognosis, and treatment.

The role of miRNAs in cervical cancer concerns many aspects of tumor cell development and survival, including sensitivity to radiotherapy.

In their study, Wang et al. [99] showed that the silencing of miR-19a-5p significantly improved the sensitivity of SiHa cells to radiotherapy by reducing proliferation, increasing apoptosis, upregulating BCL2-associated X (BAX), and downregulating B cell lymphoma 2 (Bcl-2) [99].

Similar results have been shown by Xu et al. [100], who demonstrated that both miR-19a-5p and miR-19b-5p are vastly expressed in human cervical cancer cells and are implicated in malignant HeLa and C33A cell phenotypes [100].

In addition, miR-19a-5p and miR-19b-5p have also been shown to control CUL5 levels directly and negatively in cervical cancer cells, both emphasizing their importance and that of their target genes in tumorigenesis processes [100].

**Figure 1:**
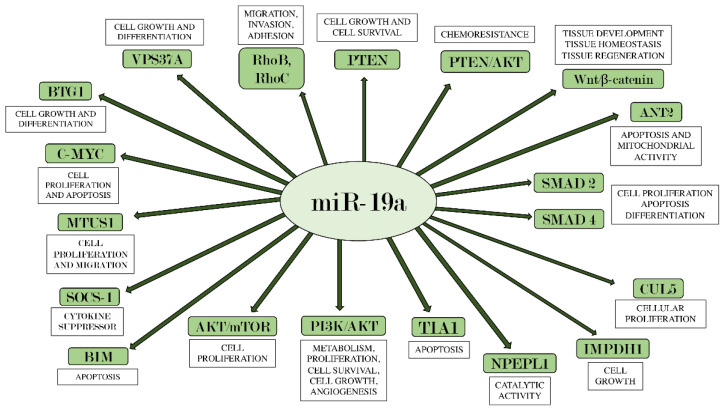
miR-19a and its main target genes. The figure summarizes the interactions between miR-19a and the target genes on which it acts, also indicating each their main biological effects.

Furthermore, it is interesting to highlight the Bcl-2 interacting mediator of cell death (BIM), an initiator of the intrinsic apoptotic pathway in both physiological and pathophysiological conditions [101]. In fact, its reduction has often been associated with tumor promotion, while its overexpression has the ability to inhibit tumor growth and resistance to chemotherapy [102]. Therefore, *BIM* has emerged as a key mediator in the regulation of tumorigenesis, as demonstrated by several studies [103,104] that indicate it as a promising target in the field of anticancer therapy. In particular, the miR-17-92 cluster has been found to suppress *BIM* expression in multiple myeloma cells [105], human ovarian cancer cells [106], and esophageal adenocarcinoma [102]. miR-19a, as a prominent component of the miR-17-92 cluster, can facilitate tumor formation, inhibiting BIM expression and promoting the proliferation of tumor cells [107]. The function of BIM, along with all other miR-19a targets described in this review, is shown in Figure 1.

**Table 1:** This table summarizes the relationship between the miR-19a isoform and its target genes in several types of cancers.

Tumor Type	Isoform of miR-19	Type of Study	Target Genes	Reference
Gliomas	miR-19a	in vitro and clinical	PTEN	[40]
	miR-19a	in vitro and clinical	RhoB	[44]
	miR-19a	in vitro, in vivo, and clinical	EPB41L5, RhoC, p-AKT	[45]
GC	miR-19a	in vitro, in vivo, and clinical	SMAD2, Wnt/b-catenin	[51]
	miR-19a	in vitro, in vivo, and clinical	CUL5	[52]
Colorectal	miR-19a	in vitro and clinical	TIA1	[57]
HCC	miR-19a	in vitro	PTEN/Akt	[108]
	miR-19a	in vitro	PI3K/Akt	[58]
Pancreatic	miR-19a	in vitro, in vivo, and clinical	RhoB	[59]
LVSCC	miR-19a	clinical	SOCS-1	[63]
Lung	miR-19a/b	in vitro and clinical	MTUS1	[66]
Osteosarcoma	miR-19a	in vitro, in vivo, and clinical	RhoB	[109]
	miR-19a	in vitro and clinical	PTEN	[110,111]
ccRCC	miR-19a	in vitro and clinical	PTEN/SMAD4	[75]
	miR-19a	in vitro and clinical	RhoB	[76]
Bladder	miR-19a	in vitro	PTEN	[112]
	miR-19a	clinical	PI3K/AKT	[78]
	miR-19a	in vitro and clinical	PTEN	[80]
	miR-19a	in vitro and clinical	RhoB	[81]
Prostate	miR-19a	in vitro and clinical	CUL5	[85]
	miR-19a	in vitro, in vivo, and clinical	BTG1	[86]
	miR-19a	in vitro and clinical	VPS37A	[87]
Myeloma	miR-19a	in vitro	PTEN/AKT/pAKT	[96]
Ovarian	miR-19a	in vitro and clinical	PTEN	[97]
Cervical	miR-19a	in vitro and clinical	CUL5	[100]

## 3. miR-19a as a Cancer Diagnostic and Prognostic Biomarker

miRNAs are highly stable in biological fluids, and their expression level changes have been associated with tumor patient’s prognosis or treatment response. Cancer tissues secrete circulating miRNAs into the surroundings, and they can be used as tools for cancer diagnosis and prognosis, and also for distinguishing tumor subtypes [113].

**Figure 2:**
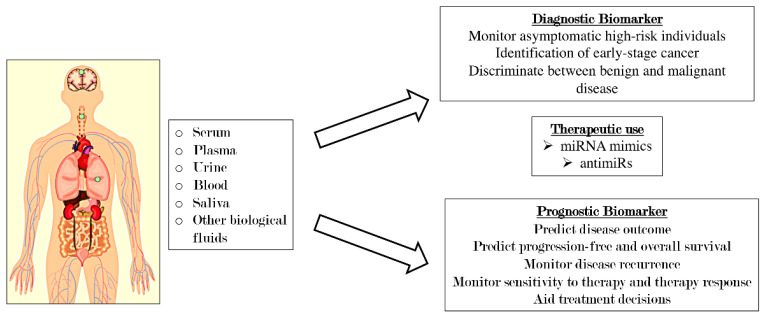
Promising clinical values of miR-19a for early cancer diagnosis, treatment, and prognosis.

Numerous researchers have developed methods to detect miR-19a in serum, plasma, urine, and other biological fluids [114], as summarized in Figure 2. Quantitative real time-PCR (qRT-PCR), microarray, and next-generation sequencing have been used to quantify the expression of circulating miRNAs [115].

Qiu et al. [116] analyzed 58 patients with undifferentiated lung cancer (experimental group) and 42 healthy volunteers (control group) and measured the expression levels of miR-19 in peripheral blood by qRT-PCR. They reported that miR-19 expression levels in the experimental group were significantly higher than those of the control group, indicating that miR-19 could be used as diagnostic markers for undifferentiated lung cancer.

In another study, Cheng et al. [117] evaluated circulating miR-19a-3p in plasma specimens obtained from 58 gastritis subjects, 54 patients with precancerous lesions, and 38 early gastric cancer (EGC) patients. Their results showed significant differences in the miR-19a-3p expression levels between EGC patients and gastritis subjects, highlighting that plasma miR-19a-3p could be a promising and noninvasive marker for the early diagnosis of GC.

Even though miR-19-5p is derived from the same precursor as miR-19-3p, few studies are reported in the literature on its role in tumorigenesis. Huang et al. [118] indicated the key role of miR-19-5p in the development and progression of CRC, via targeting TSP5, indicating that miR-19-5p could also be a useful biomarker for CRC patients, highlighting its potential implications for diagnoses and therapeutic interventions.

Other results have also been shown for osteosarcoma. Zou et al. [69] analyzed miR-19a expression patterns and investigated its clinical implication in human osteosarcomas. In this study, the miR-19a expression levels in 166 self-pairs of osteosarcoma and non-cancerous bone tissues were measured, by qRT-PCR, and in addition, the correlations between its expression, clinicopathological parameters, and patients’ prognosis were assessed. Their results reported that miR-19a expression in osteosarcoma tissues is significantly higher than in non-cancerous bone tissues, and that high miR-19a expression levels correlate with large tumor size, advanced clinical stage, positive distant metastasis, and poor response to chemotherapy. These data suggest that miR-19a could represent a novel prognostic marker for osteosarcoma patients.

The diagnostic and prognostic value of miR-19a has also been implicated in non-small cell lung cancer (NSCLC). Lin et al. [119] have reported that high miR-19a levels in serum, detected by qRT-PCR, represented an important prognostic factor for the prediction of survival and response to chemotherapy in NSCLC patients.

Furthermore, it has also been reported that miRNAs are abundant in the exosomes, which offer them stability and play an important role in tumor growth and development [120,121,122]. Several tumor-specific miRNAs, entrapped in exosomes, have been found in serum, plasma, and other biological fluids, offering an easy and early cancer diagnosis. Matsumura et al. [123] reported that exosomal miR-19a expression levels in serum were significantly higher in patients with colorectal cancer (CRC) than in healthy individuals, suggesting its possible use as a prognostic biomarker for recurrence in CRC patients.

Numerous miRNAs are currently in clinical trials as biomarkers for cancer classification and progression and as prognostic tools. Moreover, several miRNAs modulators (miRNAs mimics and antimiR) have entered the clinical trials as miRNA-based therapeutic strategies to achieve tumor regression [124]. miRNA antagonists are developed to inhibit miRNAs that acquire a gain of function in cancer disease, resulting in increased expression of the tumor suppressor genes. Examples of miRNA antagonists are antimiRs [125], antagomiRs [126], and locked nucleic acids [127].

On the other hand, miRNA mimics play a contrary role in regulating the expression of target genes, re-establishing miRNAs that show a loss of function. Therefore, the miRNA mimics approach, also known as miRNA replacement therapy, could represent a novel chance of treatment for many cancer types and stages.

## 4. Conclusions and Future Perspectives

Accumulating studies have demonstrated that miR-19a is aberrantly expressed in various tumor types, and its overexpression has been associated with cell proliferation, invasion, migration, metastasis, tumor size, stage of development, and poor prognosis.

Although several hypotheses on the role of miR-19a in cancer have been proposed, its biological function and mechanism remain unclear and further analyses to determine its mechanism in tumors are necessary, as well as the correct therapeutic approach.

Furthermore, considering that circRNAs exert critical functions in tumor progression via sponging miRNAs, future research should be conducted to better elucidate possible applications in diagnostic and therapeutic oncology.

Thus, miR-19a is predicted to be a probable candidate as both a biomarker and novel therapeutic target for diagnostic and prognostic applications.
